# Plant Metabolomics: Current Initiatives and Future Prospects

**DOI:** 10.3390/cimb45110558

**Published:** 2023-11-08

**Authors:** Sudha Manickam, Veera Ranjani Rajagopalan, Rohit Kambale, Raghu Rajasekaran, Selvaraju Kanagarajan, Raveendran Muthurajan

**Affiliations:** 1 Department of Plant Biotechnology, Centre for Plant Molecular Biology and Biotechnology, Tamil Nadu Agricultural University, Coimbatore 641003, India; sudha.m@tnau.ac.in (S.M.); rajaranji@gmail.com (V.R.R.); rohitkamble568@gmail.com (R.K.); raghu.r@tnau.ac.in (R.R.); 2 Department of Plant Breeding, Swedish University of Agricultural Sciences, P.O. Box 190, 234 22 Lomma, Sweden

**Keywords:** metabolomics, analytical tools, mass spectrometry, plant metabolomics, crop improvement

## Abstract

Plant metabolomics is a rapidly advancing field of plant sciences and systems biology. It involves comprehensive analyses of small molecules (metabolites) in plant tissues and cells. These metabolites include a wide range of compounds, such as sugars, amino acids, organic acids, secondary metabolites (e.g., alkaloids and flavonoids), lipids, and more. Metabolomics allows an understanding of the functional roles of specific metabolites in plants’ physiology, development, and responses to biotic and abiotic stresses. It can lead to the identification of metabolites linked with specific traits or functions. Plant metabolic networks and pathways can be better understood with the help of metabolomics. Researchers can determine how plants react to environmental cues or genetic modifications by examining how metabolite profiles change under various crop stages. Metabolomics plays a major role in crop improvement and biotechnology. Integrating metabolomics data with other omics data (genomics, transcriptomics, and proteomics) provides a more comprehensive perspective of plant biology. This systems biology approach enables researchers to understand the complex interactions within organisms.

## 1. Introduction

Recent improvements in plant biotechnology techniques have significantly deepened our understanding of the metabolic regulations in individual plants. Over the last two decades, sophisticated molecular omics technologies have been widely used. These include integrating high-throughput technologies using liquid chromatography–mass spectroscopy (LC-MS) and gas chromatography–mass spectrometry (GC-MS) approaches to identify new metabolic regulations in existing pathways that influence the cellular physiology, and, ultimately, the plant phenotype. Recent metabolomics initiatives have prioritized yield-related features with a focus on increasing quality. In particular, integrating metabolomics with other approaches, like quantitative genetics, transcriptomics, and genetic manipulation, has shown its crucial role in crop improvement.

Several integrated technologies and methodologies, such as methods based on mass spectrometry (MS), are employed for the large-scale exploration of highly complex plant extracts. They include GC-MS, LC-MS, NMR, MALDI, capillary-based MS, and other MS-based techniques. In addition, the emergence of genome editing tools has enabled plant biologists to perform precise and efficient targeted modification in a wide variety of plant species to identify gene functions and manipulate metabolic pathways. Notably, applying these modern tools has enabled crop improvement programs to flourish by enhancing the quality traits, including flavonoids, folate, and protein composition. This comprehensive review focuses on the latest investigations into plant metabolomics and its applications for crop improvement.

## 2. Metabolomic Platforms and Large-Scale Metabolite Databases

**Figure 1:**
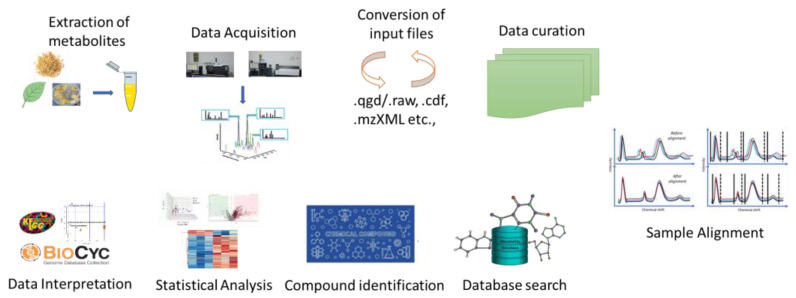
Schematic representation of metabolomics workflow.

Metabolomics is a dynamic and developing area that comprehensively understands the metabolic characteristics of biological systems. Metabolomics is the systematic study of the metabolome of cells, biofluids, tissues, or organisms, utilizing high-throughput analytical techniques to identify and measure the changes in metabolites linked with diseases. Multiple analysis techniques are required due to the complexity of the metabolome and the vast range of physiochemical properties of the metabolites. Mass spectrometry, NMR, LC-MS, and GC-MS are the most often utilized analytical platforms. These approaches enable extensive data generation and enhanced chemometric analysis, which provide basic information about the metabolites (Figure 1). In contrast to NMR, mass spectrometry’s higher sensitivity enables researchers to systematically cover the metabolome data. Due to this, researchers were able to find novel metabolic biomarkers and molecules that can aid the reconstruction of metabolic networks. Recent developments in ionization technologies, such as air pressure chemical ionization (APCI), electrospray ionization (ESI), and MALDI-TOF, have improved the accuracy of mass spectrometry [1]. Due to the large sample requirement of NMR and its lower sensitivity, its capacities to identify physical properties of ligands, binding sites on the protein, direct binding of the target protein, and the detection of protein–ligand complex structures continue to be its advantages over MS.

The GC-MS platform involves the derivatization of samples, making the compounds volatile and leaving underivatized compounds (except hydrocarbon) unnoticed during analysis. Higher sample throughput and co-eluting peak separation have been made possible by the advent of GC X GC-TOF-MS [2]. To identify both primary and secondary metabolites of higher mass, LC-MS primarily employs ESI and APCI, which are frequently utilized for targeted and non-targeted approaches [3]. In addition to these platforms, targeted metabolomics focuses on analysis of specific categories of metabolites with precise selectivity as well as on sensitivity and untargeted metabolomics studies in analyzing all detectable metabolites, including unknown compounds. Capillary electrophoresis–mass spectrometry (CE-MS) offers high-resolution separation of various analyte groups (charged, neutral, polar, and hydrophobic) [4]. MS is also coupled with FAIMS (field asymmetric waveform ion mobility spectrometry), an electrophoretic method based on ion mobility. Biological samples, such as volatile chemicals produced during bacterial growth, are detected using the FAIMS method [5]. MET-COFEA, MET-Align, ChromaTOF, and MET-XAlign are a few examples of the data processing platforms used to process the extensive data sets produced by the aforementioned high-throughput technologies [6,7,8]. Prior to the identification of chemicals, this involved baseline correction, alignment, separation of co-eluting peaks, and normalization (Figure 1). METLIN, NIST, GOLM, and other metabolome databases can be utilized to detect metabolites [9]. Additionally, utilizing web-based tools and software like MetaboAnalyst 5.0, Cytoscape 3.10.1 and statistical analysis tools, data are subjected to statistical analysis to detect the metabolites [10,11]. Locating metabolic markers linked to various traits is made easier by these analyses. Initiatives like the Plant Metabolic Network (PMN) and the Metabolomics Workbench will provide centralized databases of plant metabolites, pathways, and related information that will aid researchers in data sharing and analysis.

## 3. Role of Metabolomics in Crop Improvement

Metabolomics is a promising approach to the understanding of abiotic stress tolerance in plant species. The use of metabolomics can help in designing novel strategies to direct metabolism towards crop improvement. Metabolomics has recently been used to seek unique metabolites in plants throughout their life cycles. Crop yield loss is significantly affected by biotic and abiotic stresses [12]. The identification of specific events that activate immune sensors in plants to provide resistance, such as effector-triggered immunity, pattern-triggered immunity, and pattern recognition receptors, is necessary for the detection of invasive species. The plant produces phytohormones to provide stress resistance as soon as abiotic stress occurs. Stomatal conductance is disrupted by oxidative stress, which also activates a number of signaling systems [13]. Overall, a specific plant species with a unique gene expression profile reflects the precise composition of its metabolites. The activation of a specific metabolic network results in the synthesis of a novel bioactive compound [14]. The general steps involved, from diagnostics to metabolomics-assisted breeding for crop improvement, are shown in Figure 1.

## 4. Metabolomics and Its Regulations in Abiotic Stresses

**Figure 2:**
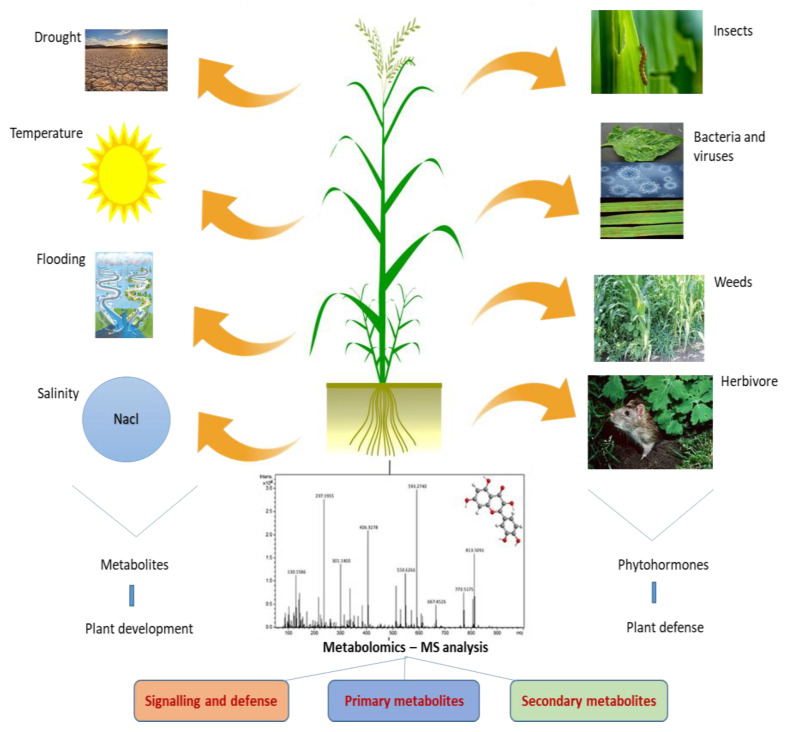
Plant metabolomics: a new era in the advancement of crop improvement.

The most promising technique for understanding the regulation of abiotic and biotic stress tolerance in plant species is metabolomics (Figure 2). In metabolomics studies, a plethora of sophisticated MS-based instruments are widely utilized to enhance the comprehension of plants’ ability to withstand abiotic stress [15]. In general, plant metabolic profiling under abiotic stressors can be performed using GC-MS. Time of flight–mass spectrometry allows for the quick and efficient discrimination and detection of a variety of metabolites in mixed samples, which is beneficial for the identification of abiotic stress-regulated metabolites [16,17,18]. Abiotic stresses drastically change plant growth and development, severely restricting plant distribution and lowering the agricultural productivity [17]. Plants experience osmotic stress as a result of altered ion concentration and homeostasis under drought and salinity stress [19]. All fundamental metabolites, including sugars, sugar alcohols, and amino acids are difficult to synthesize in plants under abiotic stressors [20].

Eight wheat cultivars were subjected to GC-MS metabolic profiling in order to gain insights into the mechanism of drought tolerance. Under drought stress, an elevated amino acid concentration was observed [21]. In 2018, Yang and colleagues [22] applied RP/UPLC-MS to conduct metabolic profiling of drought-stressed maize. The results indicated increased lipid and carbohydrate metabolism, along with an accelerated glutathione cycle. Metabolic profiling using LC-MS and GC-MS data also supported the difference in metabolite accumulation between young and mature leaves [23,24]. A GC-MS technique was used to detect increased synthesis of 4-hydroxycinnamic acid, ferulic acid, stearic acid, and xylitol in rice under drought conditions [6]

GC-MS-based metabolic profiling of rice seedlings under salt stress revealed the hyperaccumulation of key amino acids such as leucine, isoleucine, valine, and proline [25]. Comparative metabolic profiling using GC-TOF-MS in salinity-tolerant and susceptible genotypes of rice revealed higher concentrations of amino acids [26]. GC-MS based profiling under salinity stress conditions revealed elevated levels of proline, sucrose, xylose, maltose, and organic acids [27]. Another investigation on rice grown under salt stress found that it possessed less shikimate and quinate [28]. In rice, using jasmonate has been demonstrated to reduce salt damage. The jasmonate pathway is a crucial hormonal mechanism of great relevance [29]. Furthermore, metabolomics technologies have been used to investigate changes in the metabolic profiles of numerous crop plants. Furthermore, various metabolomics tools have been used to investigate changes in the metabolic profiles of numerous crop plants, including tomato, maize, barley, and wheat [30,31,32].

The synthesis of secondary metabolites is impacted by heat stress [33]. LC-MS/MS-HPLC profiling of wheat grains revealed higher amounts of sucrose during heat stress [34]. Comparative metabolic profiling of heat-tolerant and susceptible soybean genotypes showed higher concentrations of carbohydrates in the heat-tolerant genotype. Many metabolites, including arabitol, pinitol, and erythritol, were also produced in lower concentrations by these tolerant genotypes [35]. In order to observe the impacts of heat stress, metabolomics studies were also carried out for other significant crops, including tomato, maize, and wheat [36,37,38].

According to metabolic fingerprinting, tomato plants under N stress have lower concentrations of organic and amino acids [39]. A metabolic profiling technique based on UHPLC revealed that barley underwent nutrient stress-induced synthesis of organic acids, amino acids, and S-responsive metabolites [40]. Metabolic profiling of P-deficient barley exhibits lower amounts of various organic acids [41]. Similarly, P stress in nodules and roots was examined by common bean metabolic profiling [42], and low nitrogen levels in wheat were also studied [43].

## 5. Metabolite Accumulation in Biotic Stresses

Metabolomics profiling identifies significant changes in plant primary and secondary metabolites as a result of pathogen infection [44]. To activate defense mechanisms against pathogen attacks, plants adopt various strategies. It is challenging to decode the entire metabolome of a plant species because plant cells contain a diverse range of metabolites [45]. In response to biotic stressors, plants have built up a number of metabolites that function as biomarkers to control biotic stress resistance in different plants [46]. Significant amounts of benzoxazinoids (BXs), essential secondary metabolites, have been found in maize, acting as a defensive mechanism against biotic stress [47]. The identification of complex metabolic networks involved in plant–pathogen interaction is enabled by comparative metabolic profiling of diseased and healthy plants [48].

The capillary electrophoresis/time of flight mass spectrometry platform was used to investigate rice cultivar tolerance to *Rhizoctonia solani*. Upon fungal infection, higher quantities of glyceric acid, jasmonic acid, and mucic acid were generated [49]. To better understand the metabolomics of viral infection, horse gram germplasms were evaluated for tolerance to horse gram yellow mosaic virus, marking extreme groups of resistance and susceptibility [50]. GC-MS was used to pinpoint a range of biomolecules that contribute to HgYMV resistance. In the highly resistant genotype, the metabolite profile revealed a significant accumulation of three anti-virals (octadecanoic acid, diphenyl sulfone, and 2-aminooxazole), one insecticidal (9,10-secocholesta-5,7,10(19)-triene-3,24,25-triol), one antifeedant (cucurbitacin B), and six metabolites with unknown biological functions. In our other study, comparative GC-MS analyses revealed that the powdery-mildew-resistant mutant in horse gram expressed thirteen classes of unique defense-related metabolites that allowed it to withstand pathogenicity with minimum yield loss [51].

Higher accumulations of phenylalanine, glutamine, and linoleic acid were identified in gall-midge-resistant rice varieties by the GC-MS technique [52]. Similarly, GC-MS profiling showed higher amounts of carbohydrates, lipids, alkaloids, acetophenone, and xanthophylls in the bacterial-leaf-blight-resistant rice varieties [53]. Using LC-MS, GC-MS, and NMR-based metabolomics methods, the metabolomic profile of rice infested with *Magnaporthe grisea* was carried out and found to have a varied metabolomic profile. Similarly, two metabolites, namely smiglaside and smilaside, were identified in maize when examining the mechanism behind resistance to *Fusarium graminearum* [54]. Profiling of metabolites revealed the presence of polyphenols, lignin, and flavonoids in an analysis for resistance to southern corn leaf blight using FT-IR and NMR techniques [55].

## 6. Metabolomics in Assessing the Nutrients

Plants require essential nutrients for normal growth and development. Metabolites are formed in plant cells from structural units including carbon, phosphorus, sulfur, and nitrogen. Nitrogen serves as a fundamental structural unit for cellular metabolites, namely nucleic acid, amino acids, and proteins, as well as for several secondary metabolites [56]. Large numbers of metabolites like threonate, glycerate, and inositol were synthesized under a limited supply of nitrogen [57]. A comparative metabolomic analysis of the nutritional and therapeutic potential of rice grains of the traditional variety Mappillai Samba profiled the phytochemical contents of the therapeutically known traditional rice against white rice CBMAS 14065 using non-targeted GC-MS/MS. This study identified therapeutically important metabolites in Mappillai Samba [58]. Khan and colleagues used GC-MS and LC/MS technology to undertake wheat metabolomic profiling and discovered increased tyrosine, lysine, allo-inositol, and L-ascorbic acid synthesis in wheat under N stress [18]. Under N stress, increased amounts of fructose, ribulose, and lyxose were found in a wheat metabolome investigation using an integrated GC-TOF-MS technique [59].

## 7. Metabolomics in Discovering Biomarkers

Metabolomics is used in plant biology to discover biomarkers associated with specific physiological conditions or diseases. This can have applications in plant breeding and precision agriculture. An efficient method of screening was developed and validated to identify metabolic markers of several phenotypes from different environmental variations or from the availability of genomic data. An untargeted screening procedure is utilized to find biomarkers for traits with unknown biochemical mechanisms. The metabolic biomarker screening tool is employed in the identification of biomarkers for several complex traits that include yield, disease resistance, and stress tolerance.

Several metabolites have been identified as diagnostic biomarkers, such as fructose, tyrosine, glucose, glutamine, threonine, serine, and valine, in post-harvest quality trait identification of potato tubers. Metabolomics was also utilized to select progeny with desired traits from a segregating breeding population [60]. Though metabolite-based biomarkers or metabolite biomarkers have not yet been reported, metabolic biomarkers are commercially exploited. In addition, recently reported information on metabolomic profiling revealed the presence of signature metabolites.

Efforts have been made in the recent past to analyze the parental metabolomes in order to predict the power for heterosis for the selection of biomarkers. Gärtner et al. [61] identified predictive biomarkers for biomass heterosis in *Arabidopsis* and demonstrated that metabolic analysis can significantly improve the efficiency of genetic data, suggesting the complex mechanism underlying heterosis.

## 8. Metabolomics-Assisted Breeding

Metabolite profiling serves as a powerful tool for guiding the breeding process towards identification of promising traits in the early stages of selection. Genotypic variation is also assessed with the use of metabolite profiling prior to the development of molecular tools for a particular species [62].

High-throughput metabolome analysis paved the way for significant advancements in software tool design as well as instrumentation innovation during the last decade. Research fields, including biotechnology, functional genomics, precision plant breeding, and disease diagnostics benefited from the applications of metabolomics and also the move forward toward translational metabolomics [63]. Recent advancements have sped the screening process, and the incorporation of metabolomic technologies will shorten the time required to generate elite crop varieties resistant to biotic and abiotic influences. In addition, metabolomics has a greater potential for the diagnosis of metabolites and plant phenotyping [64]. Approximately 840 metabolite units were found in 524 rice varieties, having the potential to be exploited in crop-breeding strategies in the future [65].

The combination of metabolomics and association-mapping approaches showed the linkage between genomic regions of maize and kernel composition, starch content in potato, pigment content in tomato, and pro vitamin A in maize [64]. Targeted metabolomic platforms in turn benefit from several strategies because of higher mapping resolution and high allele numbers [66] The transition from single metabolite measurements to metabolomic platforms led to the developments of models that link different biochemical pathways, metabolisms, and yield related traits. The key alleles identified in crops like tomato, wheat, rice, sesame, broccoli, mustard, and Arabidopsis were utilized in metabolic engineering.

Metabolic genome-wide association studies (mGWASs) have recently emerged as a tool to elaborate the natural genetic basis of several metabolic changes in the plant metabolome. Novel candidate genes were identified in rice in an efficient manner by phenotypic genome-wide association studies (pGWASs). The identification of biomarker metabolites in *Arabidopsis* wild type and mutants used an integrative approach to metabolic profiling and resulted in hundreds of individual compounds. Identification of biomarker metabolites within metabolite protein correlation networks allowed the visualization of inherent time-dependent biological characteristics in the identification of metabolites and proteins. This serves as a promising approach toward diagnostic technology and biomarker discovery.

The location of genetic factors that determine natural variation in mapping populations led the way in identifying biomarker metabolites that reflect the genotypic and phenotypic variations in crops. These findings clearly define the way to integrate the studies of the complex regulation of plant metabolism, which can be used for traditional breeding and for metabolic engineering of agronomically important crops. In plant biology, metabolomics has a key role as a fundamental tool in systems biology research, which also has great potential for predictions and diagnostics for plant breeding and biotechnology. Intensive development of comprehensive databases will accumulate and elaborate information about metabolic networks. The correlation between the genotype and phenotype will provide rich sources for the search of new, valuable phenotypic traits and their metabolite markers.

## 9. Implications of Data Science in Plant Metabolomics

Data science has the ability to completely revolutionize our understanding of plant metabolism. In order to gain insights about plants’ development, growth, and interactions with their environment, enormous amounts of complicated data are being analyzed using techniques like machine learning, statistical modeling, and network analysis. The goal of applying data science in plant metabolomics is to demonstrate the potential for analyzing data from studies on plant metabolism using various data science methods and approaches [67].

Data science techniques are important in order to effectively address the problems caused by harmful plant metabolites and environmental concerns. These techniques involve analyzing vast, complicated datasets using statistical, computational, and mathematical tools, allowing the detection of patterns, correlations, and trends that may not be readily obvious from the raw data. Multivariate analytic tools, like principal component analysis (PCA), partial least squares (PLS), partial least squares discriminant analysis (PLS-DA), and orthogonal projections to latent structures (OPLS), are important tools among the statistical techniques and software programs. These methods can aid in locating metabolite data patterns that might be connected to particular biological elements, such as treatment conditions or genetic variation. In response to biotic and abiotic challenges, a thorough overview of the many analytical techniques that can be employed to identify alterations in plant metabolomics was provided by [68]. To gain a more thorough understanding of plant stress responses, the author emphasized the significance of integrating various forms of data, such as transcriptome and proteomic data, with metabolite data. To research plant metabolites with the involvement of more metabolomic pathways and their effects on the environment, metabolomics uses a variety of data science techniques and modeling.

**Figure 3:**
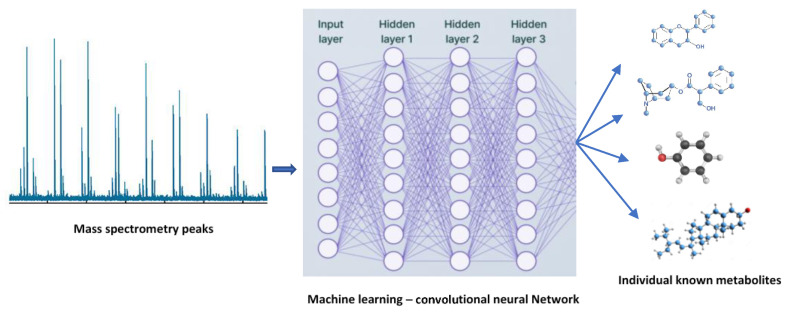
Applications of machine learning in plant metabolomics.

The use of artificial intelligence (AI) for the classification of plant metabolites and plant metabolism is still in its infancy. However, there are some possible uses for AI in plant metabolomics. To find new secondary metabolites and their functions, for instance, large-scale metabolomic datasets can be analyzed using AI algorithms. AI algorithms are effectively used to analyze large-scale metabolomic datasets for novel secondary metabolite identification (Figure 3). Additionally, machine learning algorithms can be used to categorize and forecast the roles of various metabolites based on their structural properties and other variables [67]. This can improve our comprehension of the roles played by various plant metabolites during plant development, defense, and environmental interactions. In turn, the use of data science techniques predicts the role of plant metabolism in influencing the environmental factors, as well as in improvising the strategies in plant breeding programs.

Furthermore, it is possible to gather a wealth of information on plant metabolomic studies. Integration of data sources, like remote sensing and high-throughput phenotyping, facilitates the accuracy and efficient modeling of complex biological systems.

A convolutional neural network (CNN) was used in classifying spikes and spikelets in images of wheat to interpret plants’ development [69]. Also, a deep neural network was developed to detect pests and diseases in tomatoes [70]. Additionally, interdisciplinary collaboration between plant scientists and data scientists will enable the usage of AI in agricultural research into data integration for crop improvement.

## 10. Metabolomics for Plant-Microbe Interactions Research

Various strategies are being used to investigate the relationships between microbial species and the plants in which they live. In comparison to genomic and transcriptomic techniques, the application of metabolomics and its tools in plant–microbe interaction investigations are largely underutilized. Metabolomics not only gives a comprehensive view of the metabolic pathways involved in plant–microbe interactions, but also lights up the underlying mechanisms of host and microbe interactions. *Arabidopsis*, plant growth-promoting bacteria (PGPB), and other bacteria communicate with one another, providing an effective instance of the integration of several forms of omics data [71].

Despite the large number of studies that have been published utilizing metabolomics techniques, research in this subject is still in its early stages, with a constantly evolving methodology. Organic acids were the principal released metabolites absorbed by the bacterial enrichment in the substrate [72]. Flavonoids were identified in root exudates that trigger bacterial nod genes and initiate nodule development [15]. In another study, untargeted metabolomics were used to identify a number of lipid indicators of *Plasmopara viticola* inoculation in grapevines [73]. Another metabolomic study on the interaction of several maize genotypes with two nitrogen-fixing PGPB species identified that plant metabolites were altered by bacterial nitrogen fixation [74].

Metabolomics is an appropriate method for studying complex biological interactions within the rhizosphere. Its application to the study of beneficial plant–microbe interactions lags behind other omics approaches, despite the fact that it offers many opportunities to broaden our understanding of the underlying mechanisms of beneficial plant–microbe interactions [75]. The use of metabolomics to study plant–microbe interactions has several challenges, including determining the origin of the metabolites studied, revealing the metabolic complexity of two or more interacting organisms, and integrating metabolome data with other omics approaches.

## 11. Interrelationship between Different Omics

**Figure 4:**
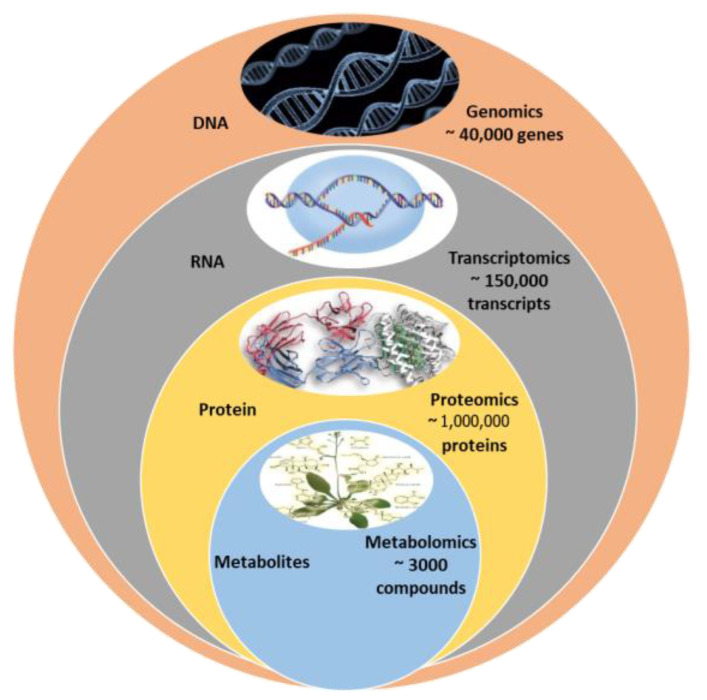
Potential of metabolomics over other omics.

Metabolomics is significantly smaller than proteome and genome, making it relatively easier for data processing and analysis. Only around 3000 metabolites are often used in the major metabolic pathways (Figure 4). The knowledge of metabolic QTLs (mQTLs) about metabolic networks controlling the complex mechanisms in metabolomics has a potential role in metabolomics-assisted breeding to develop elite cultivars for better quality and yield, providing a complete understanding of quantitative genetics [76]. Metabolic profiling identifies single-nucleotide polymorphism (SNP) markers or mQTL mapping analysis for candidate gene finding by bridging the genotype–phenotype gap. Metabolic markers are powerful tools for identifying agronomic features and investigating the metabolic mechanisms underlying diverse phenotypes [77]. The mQTLs technique dissects the integrated study of gene expression and metabolite profiles to establish a relationship between the phenotype and the genotype [76].

Because of advancements in next-generation sequencing (NGS), mQTLs for candidate genes can now be found using ultra-high-density maps [78]. Candidate genes influencing secondary metabolite production can be identified using multi-omics technologies combined with reverse and forward genetics methodologies [79]. Further, the whole metabolome uncovers population genetics with metabolomic profiling and many mQTLs have been identified in flag leaf and germinating seed across 12 chromosomes [80]. In a comparative metabolomic investigation of two rice cultivars, 19 metabolites were found on 23 loci, indicating a significant intersection of genetic regulation in distinct cells [80]. Similar reports have been found for tomato [81], maize [76,78] and potato [82]. Over 700 different metabolic characteristics were revealed in mQTL analysis of back-crossed inbred lines of rice cultivar [83].

An mQTL study of barley recombinant inbred lines (RILs) under drought stress revealed 98 metabolites. Certain stress-responsive metabolites, such as sinapic acid, ferulic acid, and flavones, act as antioxidants and regulate gene expression and protein function under stress [84]. Templer and his coworkers examined the metabolic response of barley under drought stress. Approximately 57 metabolites and unique mQTLs, namely succinate, glutathione, and -tocopherol, were identified, indicating a molecular basis for barley breeding with greater drought tolerance [85]. Metabolite profiling and genetic study of glucosinolate synthesis in *Brassica napus* revealed 105 mQTLs associated with glucosinolate biosynthesis in leaves and seeds [86]. Over 679 secondary mQTLs linked with environmental stress tolerance were identified by dissecting out the genomic regions linked with synthesizing secondary metabolites in wild and introgression lines (ILs) of tomato, [87] and tomato mQTL analysis was performed in a similar population [88]. mQTL mapping is a powerful tool for identifying traits linked with stress susceptibility. Metabolomic profiling in 179 double-haploid wheat lines using LC/MS resulted in the identification of 558 secondary metabolites, including phenylpropanoids, flavonoids, and alkaloids [89]. The RILs of tomato seeds were profiled metabolically using GC-TOF/MS to identify the interaction between seed metabolism, environment, and genetics in identifying metabolites [90]. Plant metabolite environment interactions can be clearly understood by coupling metabolomics with high-throughput phenotyping technologies.

Numerous studies have also identified mQTLs that regulate biotic interactions in plants. With the advent of modern sequencing technology, many plant genomes have been sequenced, frequently using mQTL analyses in agricultural crops. The host-pathogen candidate genes are identified by mQTL mapping, which also analyzes the pathways that govern the resistance mechanisms in plants. A holistic understanding of plant biology will be provided only by integrating metabolomic data with other omic approaches like genomics, transcriptomics and proteomics. Apart from the above studies, integrating metabolomics data into systems biology models will certainly provide a comprehensive understanding of how metabolites interact within cellular networks, leading to predictive models of plant behavior.

## 12. Concluding Remarks and Future Prospectives

In conclusion, plant metabolomics has emerged as a powerful tool for unraveling the complexity of plant metabolism and understanding its diverse functions. It has provided valuable insights into plant biology, facilitated the discovery of novel bioactive compounds, and offered opportunities for improving crop traits and addressing global challenges in agriculture. Linking metabolite profiles with specific biological functions will help to elucidate the roles of various metabolites in plant growth, development, and responses to environmental cues. As technology and analytical methods continue to advance, plant metabolomics is expected to contribute even more significantly to our understanding of plant systems and the development of innovative solutions for a sustainable future.

## Data Availability

No new data were created or analyzed in this study. Data sharing is not applicable to this article.
